# Prevention of sarcopenia in patients with obesity after bariatric and metabolic surgery: The effect of programmed training on the muscle tissue and anthropometric functions—A randomized controlled trial (SarxOb study protocol)

**DOI:** 10.17305/bjbms.2022.7786

**Published:** 2023-03-16

**Authors:** Marek Bužga, Matej Pekař, Jaroslav Uchytil, Veronika Horká, Jan Malůš, Dominik Vilímek, Zdeněk Švagera, Petr Kutáč, Pavol Holéczy

**Affiliations:** 1 Department of Physiology and Pathophysiology, Medical Faculty, University of Ostrava, Ostrava, Czech Republic; 2 Department of Physiology, Medical Faculty, Masaryk University, Brno, Czech Republic; 3 Vascular and Miniinvasive Surgery Center, Hospital AGEL Trinec-Podlesi, Trinec, Czech Republic; 4 Human Movement Diagnostic Center, University of Ostrava, Ostrava, Czech Republic; 5 Department of Cybernetics and Biomedical Engineering, Faculty of Electrical Engineering and Computer Science, VSB - Technical University of Ostrava, Ostrava, Czech Republic; 6 Institute of Laboratory Medicine, Medical Faculty, University of Ostrava, Ostrava, Czech Republic; 7 Department of Surgery, Center of Bariatric Medicine, Hospital AGEL - Ostrava - Vitkovice, Ostrava, Czech Republic; 8 Department of Surgical Disciplines, Medical Faculty, University of Ostrava, Ostrava, Czech Republic

**Keywords:** Sarcopenia, exercise, bariatric, metabolic, surgery

## Abstract

Obesity is a serious metabolic disease that significantly increases cardiovascular risks and other health complications. Sarcopenia is an independent risk factor for morbidity and mortality in patients suffering from obesity that increases the health risks and is associated with cardiac, respiratory, and other diseases. Bariatric and metabolic surgery (BMS) leads to significant changes in body composition. Our pilot study showed that bariatric patients are at risk of sarcopenia after BMS. This finding resulted in a hypothesis that an exercise plan in the experimental group will lead to postural stabilization and a lower decline in muscle homotopy, further leading to a greater reduction in fat mass and a positive effect of exercise on skeletal muscle volume and strength and endocrine-metabolic function. The aim of the present study is to determine the effect of programmed aerobic and strength training on muscle function, volume, and morphology in patients after BMS. The study is a single-center, randomized clinical trial after sleeve gastrectomy focused on muscle tissue. The experimental group will perform targeted physical activity once a week for 12 months and the training plan will include anaerobic and aerobic components. Magnetic resonance imaging of skeletal muscles will be correlated with the values of densitometry examination and changes in body composition, certain blood parameters of myokines, biomechanical analysis of movement abnormalities, and behavioral and dietary counseling. This study will address the research questions about the effect of programmed training on muscle tissue and muscular functions after BMS.

## Introduction

Over the past two decades, obesity has become a serious global health problem. In Central and Eastern Europe, the prevalence of obesity stands as a forefront issue in all epidemiological studies [1, 2]. Since the end of the 1990s, surgical treatment (BMS) has been shown to be the most effective treatment for obesity [3]. Unlike conservative treatment (lifestyle changes, balanced dietary intake, physical activity, and pharmaceutical therapy), which fails in more than 80% of patients over the long term, metabolic surgery results in long-term success in more than 80% of patients [4].

Patients with obesity are also at risk for comorbidities, such as type II diabetes, hepatic steatosis, hypertension, sarcopenic obesity, and others. The combination of low muscle mass and low strength with increased fat mass may further aggravate, metabolic disorders and physical disability with a synergistic effect [5]. Common guidelines issued by the American Society for Metabolic and Bariatric Surgery, the Obesity Society, and the American Association of Clinical Endocrinologists recommend that postoperative patients adhere to a healthful lifestyle that includes exercise for at least 30 min•per day [6].

Our pilot study published in 2020 showed that after BMS, patients are at risk of developing sarcopenia. BMS leads to significant changes in body composition. Significant fat loss is also followed by unintended muscle loss. The combination of low muscle mass and strength with increased fat mass infiltration, known as sarcopenic obesity, may have a synergistic effect, further aggravating of metabolic disorders and physical disability. A key pathophysiological mechanism in the etiology of this problem is the inflammatory activity of the excessive fat tissue. Moreover, sarcopenia in obese subjects is associated with a higher risk of hypertension, arterial stiffness, dyslipidemia, insulin resistance, knee arthritis, and osteoporosis, increasing the risk of falls and fracture and difficulties with physical function. It was found that the lack of physical activity typical of these patients contributes to this effect [7].

Therefore, there is a need for further research to elucidate the pathophysiological mechanism responsible for sarcopenic obesity and the effect of exercise on muscle tissue in patients after BMS. Previous studies in this area have focused on the effect of exercise and weight loss after BMS on improving comorbidity parameters [8]. However, most of these studies had an inadequately described exercise plan and definition of exercise intervention. Currently, there are only two studies that have investigated physical activity, exercise plans and intervention in patients in the postoperative period in detail [9, 10].

The present study plans to investigate the relationship between muscle activity in patients with obesity and the effect of long-term exercise, the effect of weight reduction on muscle tissue, the pathophysiology of myokines affecting carbohydrate metabolism, and finally, the effect of weight reduction on musculoskeletal function. Such a comprehensive pathophysiological study using rigorous methods to assess the pathophysiology of muscle tissue, the musculoskeletal system, and the effect of exercise has not been carried out. This research is especially important given the expected increase in the prevalence of obesity and sarcopenia over the next 20 years [11].

### Hypothesis and research aims

#### Hypothesis

- The inclusion of an exercise plan in the intervention group will lead to the stabilization of posture and a decrease in muscle homotopy compared to the non-exercise plan group.
- The effect of exercise will result in a greater reduction in fat mass and a positive effect on skeletal muscle volume, strength, and endocrine-metabolic function.
- Regular exercise in the intervention group will increase gait stability and reduce the risk of falls compared to the non-exercise plan group.

#### Aims of the prospective study

- To determine the effect of programmed aerobic and strength training on muscle function, volume, and morphology in patients after BMS.
- To determine the effect of weight reduction on the articular cartilage of the knee joint and to determine the effect of weight reduction on biomechanical movement parameters associated with osteoarthritis of the weight-bearing lower limb joints.
- To determine the changes in serum levels of biochemical nutritional parameters associated with skeletal muscle.
- To determine the improvement from baseline quality of life as measured by the Impact of Weight on Quality of Life questionnaire (IWQOL) and the 36-Item Short Form Survey (SF-36).

## Materials and methods

This is a prospective randomized clinical trial aimed at examining and assessing changes in muscle, metabolic muscle processes, and locomotion after BMS over the course of 18 months. Participants will be randomly assigned to the experimental or control groups. Blinding of the study groups is not possible due to the type of intervention (exercise program). The patients will undergo the surgical procedure at the Center of Bariatric Medicine, Hospital AGEL – Ostrava-Vitkovice, and the study visits will be performed at the Human Movement Diagnostic Center at the University of Ostrava, Czech Republic.

### Sleeve gastrectomy

Sleeve gastrectomy will be carried out using laparoscopic surgery as described in our previous study [12]. The procedure is expected to be the same in the two groups (control vs. experimental).

### Control group (usual care)

Participants in both groups will undergo a postoperative follow-up routinely prescribed at the Center of Bariatric medicine, according to the international standards [13]. During the first four weeks, all patients will maintain a semi-liquid diet. Medical consultations will be scheduled 1, 3, 6, 9, 12, and 18 months after surgery, with special attention paid to nutritional status. Usual care after BMS includes regular clinical checks by specialists (surgeon, nutritional specialist), as well as a set of recommendations for physical activity. The patients after surgery have the option to exercise in the swimming pool, on an exercise bike and treadmill or using other fitness machines at the gym. However, the exercise is not mandatory. After the end of the study, control patients will be offered the same standard exercise intervention regimen as those in the experimental group.

### Experimental group (usual care + supervised exercise)

**Figure 1.**
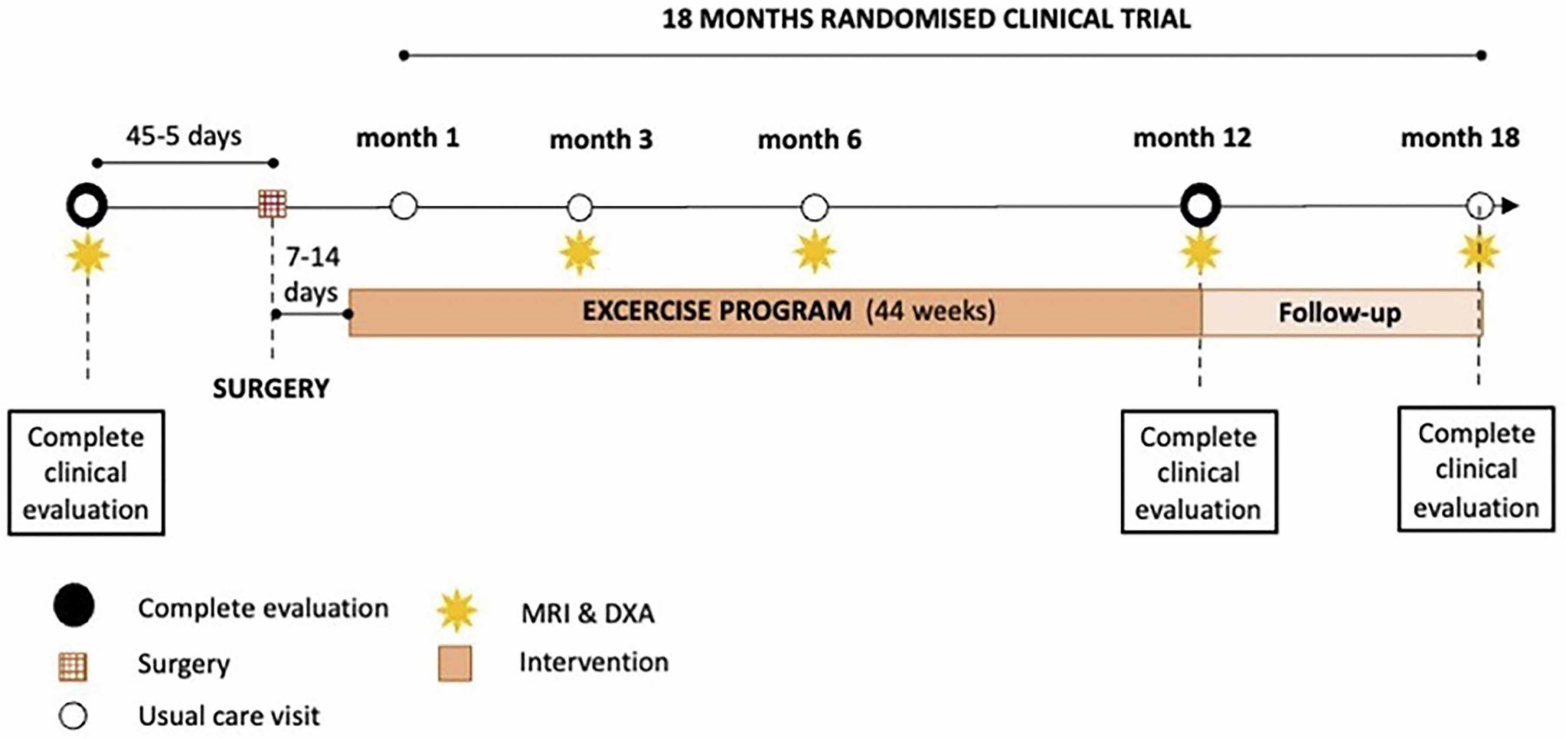
**Timeline of interventions and follow-up of participants enrolled in the study.** The figure represents the study measurements during specific intervals that are shown chronologically.

The experimental group will perform targeted physical activity once a week for 12 months under the supervision of a specialized fitness trainer. The optimal frequency of physical activity is 2–3 times a week. However, our primary goal is to provide a basic database of exercises and to check their correct execution. We strive for as feasible study design. Considering the different times of entry into the study of each participant, it is not possible (economically and time-wise) to require a controlled physical activity session more than once a week. Each participant in the experimental group will undergo 12 months of targeted physical activity and six follow-up measurements over 18 months (Figure 1).

Targeted physical activity will be performed under the supervision of a specialized fitness trainer. The training plan will be divided into a total of eight phases including a 5 min warm-up (treadmill), 5 min of breathing exercises, a 15–30 min strength component (resistance bands and dumbbell exercises), 10–20 min of aerobic activity (exercise bike and treadmill), and a 10 min of final stretching. The estimated training time is 50–60 min, never exceeding the 60-min limit. The duration of the workout will depend on the phase, which is focused on mobility and compensatory exercises, strengthening postural muscles with body weight, aerobic endurance, and strengthening smaller and then larger muscles. A precise schedule of specific exercises will be created for the intervention and controlled by the number and frequency of repetitions, which will be limited by time. The individual load will be determined by resistance bands (5, 10, and 15 kg) and weights of 7.5 and 12.5 kg. The protocol for the overall exercise plan for the experimental group is provided in Table 1.

**Table 1:** Supervised exercise protocol. Targeted physical activity once a week for 12 months with a total of eight phases, including a warm-up (treadmill), breathing exercises, a strength component (resistance bands and dumbbell exercises), an aerobic portion (exercise bike and treadmill), and a final stretching

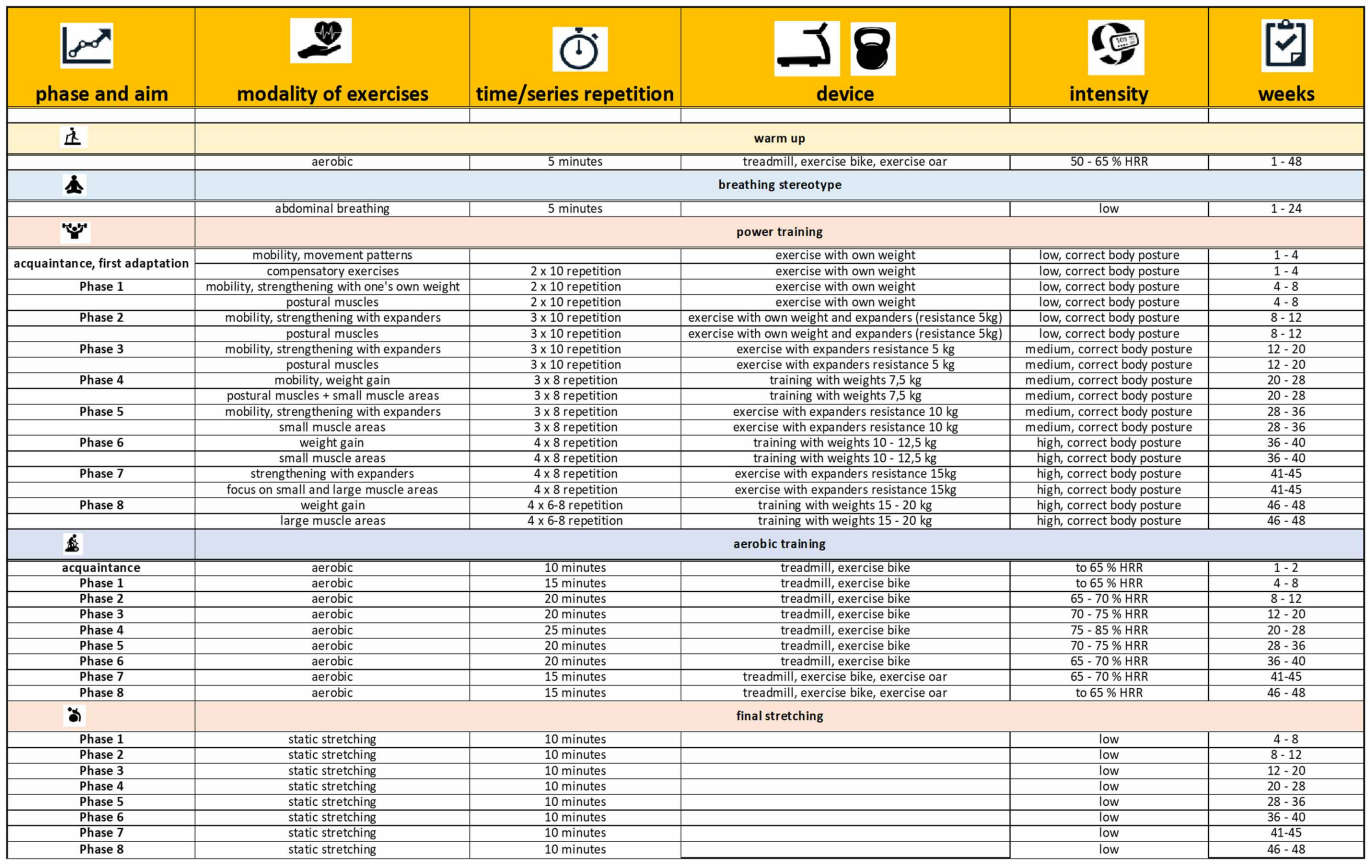

### Study visit and follow-up

During the follow-up period, patients enrolled in the study will be monitored by a multidisciplinary team that includes bariatric surgeons, endocrinologists, nutritionists, psychologists, and physiotherapists. Patients fulfilling the inclusion criteria and without exclusion criteria characteristics (Table 2) will be offered to participate in the study. Before inclusion, all patients will have to read and sign the informed consent form. The participants will have follow-up clinic visits specific to the study at 45 days (or fewer) before the surgical procedure (baseline visit) and follow-up clinic visits at 1, 3, 6, 12, and 18 months after the surgical procedure (Table 3 and Figure 1).

**Table 2:** Study inclusion and exclusion criteria. Patients fulfilling inclusion criteria and without exclusion criteria characteristics will be offered to participate in the study. There will be 20 patients in each group (control vs. experimental)

**Inclusion criteria**	**Exclusion criteria**
Age 18–65 years at screening	Diagnosis of type 2 diabetes less than six months before screening
Body mass index 35–50	Ongoing systemic infection
Gender: female/male	History of or suspected gastrointestinal disease (e.g., cirrhosis, inflammatory bowel disease)
Ability to understand the content of the informed consent form	Chronic pancreatitis
Patient resides within 100 km of the center	Chronic liver disease of any cause
**Tolerated comorbidities**	Poorly controlled psychiatric disease (e.g., ongoing major depression, schizophrenia, borderline personality, suicidality, and psychosis)
Treated hypertension	History of eating disorder within the past five years
Treated dyslipidemia	Pre-existing severe comorbid cardio-respiratory disease (e.g., congestive heart failure, cardiac arrhythmia, coronary artery disease, chronic obstructive lung disease, and pulmonary embolism)
	Uncontrolled hypertension (systolic blood pressure >150 mm Hg or diastolic blood pressure >100 mm Hg)

**Table 3:** The flowchart of interventions and follow-up of participants enrolled in the study. This table depicts the schedule of interventions at baseline and follow-up

	**Baseline**	**Follow-up**				
	**<45 days before surgery**	**Month 1**	**Month 3**	**Month 6**	**Month 12**	**Month 18**
Clinical assessment	x	x		x	x	
Screening and safety exam	x	x	x	x	x	x
MRI and DXA	x		x	x	x	x
Biomechanics exam	x		x	x	x	x
Psychological assessment	x				x	
IWQOL; SF-36	x	x	x	x	x	x
Nutrition consultation	x	x	x	x	x	x

MRI: Magnetic resonance imaging; DXA: Dual-emission X-ray absorptiometry; IWQOL: Impact of weight on Quality of Life Questionnaire; SF-36: 36-Item short form survey.

### Data collection

*Medical history:* At each visit, the investigator will record details of medical and surgical history and their changes, perform medication review, and draw a blood sample to perform standard laboratory testing for the following:

1. Glucose, glycated haemoglobin, alkaline phosphatase, total calcium, phosphate, parathyroid hormone, total protein, albumin, aspartate amino transferase, alanine amino transferase, gamma glutamyl transferase, and creatinine. Analyses will be performed on Atelica Solution, Siemens, USA.
2. After completing the evaluation of these analytes, serum (plasma) aliquots will be frozen at −80 ^∘^C until further analysis of other selected parameters, such as myokines (FGF21, IL15, IL8, and LIF). Serum levels of target hormones and cytokines will be measured on a Bio-Plex^®^ MAGPIX™ instrument (BioRad, Hercules, CA, USA) with the MILLIPLEX MAP Human Gut and Metabolic Hormone Panel (Merck KGaA, Darmstadt, Germany). Hormones and cytokines in fat tissue from all patients will be analyzed during the same testing session.

### Magnetic resonance imaging (MRI) and dual-emission X-ray absorptiometry (DXA)

*MRI:* Data will be measured before and 3, 6, 12, and 18 months after BMS on a 1.5 T Siemens Magnetom Sempra Scanner (Siemens, Erlanger, Germany). A 12-channel receiving knee coil will be used to acquire the images and MR relaxometry T2 mapping will be performed on the knee cartilage. The thigh muscle will be scanned using a six-channel body coil with T1, T2, and Dixon sequences to image adipose tissue. The imaging parameters will be as follows: repetition time/echo time ((TE)/$\Delta $TE), field of view (mm^3^), acquired voxel size (mm^3^), reconstructed voxel size (mm^3^), and flip angle (degrees). Data will be acquired during one breathhold. Proton density fat fraction (PDFF) maps for the determination of muscle fat content will be reconstructed directly on the imager console after image acquisition using vendor-specific software. Skeletal muscle analysis will use an in-house tool written in MATLAB (The Mathworks, Natick, MA, USA). Cross-sectional areas of the femoral skeletal muscles will also be obtained. MRI muscle fat infiltration will be assessed by mean radiodensity in Hounsfield units (HU) and proton density fat fraction (MRIPDFF), respectively (Tables 4 and 5).

**Table 4:** Knee MRI parameters. The table depicts default MRI sequence parameters for the knee

**Sequence**	**TR/TE (ms)**	**Flip Angle (∘)**	**Slice Thickness (mm)**	**FOV (mm^2^)**	**Matrix Size**	**Bandwidth (Hz/pixel)**	**No. of Slices**	**Acquisition Time (min:s)**
Axial PD TSE FS	4510/27	150	3	148×148	256×198	142	30	2:08
Coronal T1 SE	490/12	150	3.2	160×160	320×223	150	28	1:36
Coronal PD TSE FS	5760/25	150	3	160×160	320×235	145	35	2:08
Sagittal T2 3D-DESS WE	18/7.05	25	0.6	150×150	256×230	260	160	3:41
Sagittal PD TSE FS	4250/26	150	3	160×160	320×212	140	30	2:09
Sagittal T2 MAP	1690/12, 24, 36, 48, 60	180	3	160×160	256×256	230	18	3:55

TSE: Turbo spin echo; SE: Spin echo; PD: Proton density; FS: Fat-sat; DESS: Double echo steady state; WE: Water excitation; TR: Repetition time; TE: Echo time; FOV: Field of view.

**Table 5:** Thigh MRI parameters. The table depicts default MRI sequence parameters for the thigh

**Sequence**	**TR/TE (ms)**	**Flip Angle (∘)**	**Slice Thickness (mm)**	**FOV (mm^2^)**	**Matrix Size**	**Bandwidth (Hz/pixel)**	**No. of Slices**	**Acquisition Time (min:s)**
Coronal T1 TSE	1120/12	150	4	299×400	384×288	150	35	4:55
Axial T1 TSE	874/11	150	4	320×240	240×320	178	50	3:34
Axial PD TSE FS	3000/28	150	5	250×250	320×256	111	28	4:38
Axial T2 TSE	6500/59	150	5	250×250	320×256	200	28	2:38
Axial T1 VIBE Dixon	6.74/2.40, 4.35	10	3	217×290	288×216	500	88	5:19

TSE: Turbo spin echo; PD: Proton density; FS: Fat-sat; VIBE: Volumetric interpolated breath-hold examination; TR: Repetition time; TE: Echo time; FOV: Field of view.

*DXA:* Body composition will be assessed in all participants using the DXA method (Discovery A; Hologic, Waltham, MA, USA). The following parameters will be monitored: fat mass (kg), fat (%), estimated visceral adipose area (EST VAT; cm^2^), lean body mass (LBM; kg), appendage lean mass index (ALMI; kg/m^2^), and lean mass index (LMI; kg/m^2^). The densitometer will be calibrated according to the manufacturer’s recommendations and instrument precision will be established.

Body height (BH) and body mass (BM) will be measured as input parameters for bone densitometer software using an InBody BSM 370 stadiometer (Biospace, South Korea).

### Biomechanical analysis of movement

The kinematic analysis will be performed using 10 Qualisys Oqus infrared cameras (9× Oqus 700+ and 1× Oqus 510+, Qualisys, Inc., Gothenburg, Sweden) with a scanning frequency of 240 Hz, which will then be synchronized with three Kistler dynamometer platforms (Kistler 9286AA, 9281CA and 9287CCAQ02, Kistler Instruments AG, Winterthur, Switzerland). Wireless photocells (OPZZ, EGMedical s.r.o., Brno, Czech Republic) placed at a distance of 3 m apart will be used to control the walking speed of the test subject while passing through the force platforms.

### Monitoring of physical activity and dynamometer test

During the study, patients will have their physical activity monitored for 12 months using a Fitbit Charge 4 personal monitor (San Francisco, CA, USA). Furthermore, the force of a handshake will be monitored using a Kern MAP 130K1 dynamometer. The dynamometer test measuring 3× maximum hand pressure will be performed for both dominant and non-dominant upper limbs.

### Behavioral and dietary counseling

Behavioral and dietary counseling will be provided prior to enrollment. NutriPro software (Fitsport-komplex, Czech Republic) will be used for nutritional screening and evaluation. Subjects will be counseled to follow a regimen of routine vitamin and mineral supplementation. Prior to each scheduled follow-up, participants will be e-mailed a personal link to complete online questionnaires via Qualtrics XM to monitor their quality of life and physical activity. These are calibrated questionnaires SF 36, IWQOL, and the International Physical Activity Questionnaires (IPAQ). In addition, questionnaires will be linked to dietary habits and nutritional diary of the last three days.

### Ethical statement

The study has been designed as a prospective randomized study, which conforms to the principles and guidelines of the Helsinki Declaration, and good clinical practice and has been approved by the Ethical Committee of the Hospital AGEL Ostrava-Vitkovice, Czech Republic (no. EK/243/2020). The clinical trial registration number on Clinicaltrials.gov is NCT04617392.

### Statistical analysis

Power analysis calculation data are from the sarcopenia pilot study [7]. Estimation of the sample size for the evaluation of the mean difference in paired data was set at a significance level alpha of 5% and test power (1-beta) of 90%. Using the mean difference between ALMI and LMI as set parameters, the desired sample size was determined to be 22 individuals. Anticipating a follow-up loss of up to 20%, we plan to enroll a total of 40 patients (20 per group). Stata v.13.1 or higher (StataCorp LP, College Station, TX, USA) will be used to conduct the analyses. The statistical significance will be set at *P* < 0.05.

## Discussion

Sarcopenic obesity is a concurrence of muscle loss and body fat increase [14], including reduced baseline metabolic rate, decreased mitochondrial number and volume, and increased oxidative stress [15]. This pathophysiological sarcopenia development can be modulated by appropriate physical activity [16]. A structured exercise program is a feasible and effective adjunct therapy for BMS patients that results in additional cardiometabolic benefits compared to patients experiencing weight loss induced by BMS alone [17]. An additional research topic addressed in the present study might include the description of pathophysiological mechanisms and answering the question of how and whether exercise or physical activity can overcome the “metabolic adaptation” or decreased energy expenditure that occurs with surgery-induced weight loss and have an impact on the overall daily energy balance [18].

Compared to other studies on intervention after bariatric sleeve resection, the present study will be enriched by multidisciplinary data collection. Data from magnetic resonance, biomechanical analysis, DXA, dynamometer readings, nutritional habit analysis, biochemical analysis, monitoring of physical activity using questionnaires and Fitbit bracelets, and quality of life standardized questionnaires will help to investigate sarcopenia in bariatric patients.

In all individuals, regardless of the intervention, we are primarily expecting improvements in quality of life, changes in walking patterns, improvements in eating habits, and a reduction in total energy intake and fat and muscle mass. In individuals with intervention compared to those in the control group, a reduction in the risk of sarcopenic obesity, higher total protein albumin and pre-albumin levels, lower levels of fasting glucose and glycated hemoglobin, maintenance of a higher physical activity over a long period of time, maintenance and an increase of muscle strength, and an increase in high-density lipoprotein cholesterol levels are expected.

Radical weight reduction has been shown to have a positive effect on the biomechanical parameters of gait, which are associated with a positive effect on knee osteoarthritis [19]. However, changes in biomarkers Coll2-1 and fibulin-3 that are related to knee cartilage breakdown have not been observed [20]. Further research will clarify the relationship between weight loss and knee cartilage osteoarthritis.

## Conclusion

There is a long-standing opinion on the effect of BMS on weight loss and the effect of metabolic interventions on diabetes. However, the risk of muscle loss has not been investigated despite the fact that the prevalence of sarcopenic obesity has a fairly significant impact on health status after surgery. Recent data have shown that the reduced muscle tissue volume affects wound healing, patient mobilization, and glycaemic control after surgery. There are no current recommendations for the prevention of sarcopenia, and movement is only recommended to patients without the need for follow-up rehabilitation. The proposed study should address the research questions about the effect of programmed training on muscle tissue and anthropometric functions after BMS, as well as the effect of targeted exercise on quality of life and concomitant comorbidity improvement.
